# Microwave-Assisted Synthesis of CdS-MOF MIL-101 (Fe) Composite: Characterization and Photocatalytic Performance

**DOI:** 10.1021/acs.inorgchem.4c02104

**Published:** 2024-10-07

**Authors:** Armando Ramos Corona, Jorge Rodríguez López, Ricardo Rangel Segura, Mónica Monserrat Martínez Garcia, Eduardo Flores, Geonel Rodríguez Gattorno, Juan José Alvarado Gil

**Affiliations:** † Investigador Posdoctoral por México, Departamento de Física Aplicada, Cinvestav-Unidad Mérida, Carretera Antigua a Progreso Km. 6, Mérida, Yucatán 97310, México; ‡ Instituto Tecnológico del Valle de Morelia, Ciencias Básicas, Carretera Morelia Salamanca Km 6.5, Morelia, Michoacán 58100, México; § División de Estudios de Posgrado de la Facultad de Ingeniería Química, Universidad Michoacana de San Nicolás de Hidalgo, Gral. Francisco J. Múgica S/N, Ciudad Universitaria, Morelia, Michoacán 58030, México; ∥ Departamento de Física Aplicada, Cinvestav-Unidad Mérida, Carretera Antigua a Progreso Km. 6, Mérida, Yucatán 97310, México

## Abstract

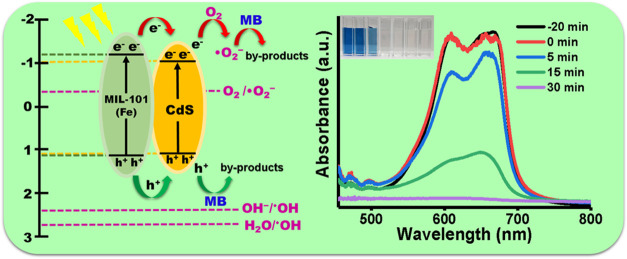

The present study outlines the synthesis of cadmium sulfide-metal–organic framework (CdS-MOF) MIL-101 (Fe) heterojunctions achieved via fast microwave-assisted reactions. Thus, different CdS-MOF MIL-101 (Fe) ratios were prepared to study their effectiveness as photocatalysts. These compounds were employed in the photocatalytic degradation of methylene blue (MB) under UV and visible irradiation. Structural, morphological, textural, compositional, and optical properties of the synthesized compounds determined by X-ray diffraction (XRD), scanning electron microscopy (SEM), X-ray photoelectron spectroscopy (XPS), UV–vis spectroscopy, Raman spectroscopy, and Fourier transform infrared (FTIR) spectroscopy were utilized to characterize the structural, morphological, textural, compositional, and optical properties. Electrochemical impedance spectroscopy (EIS) was also employed to determine photovoltage and photocurrent densities. The resulting valence band offset (*V*_fb_) and band gap energy values were utilized to construct an energy band scheme. Our research revealed that the CdS-MOF MIL-101 (Fe) heterojunction enhances the efficiency of electron–hole pair separation, thereby mitigating charge carrier recombination effects. Moreover, the type I electronic band structure established an efficient reaction mechanism, effectively suppressing the recombination of photogenerated electron–hole pairs. The photocatalytic system demonstrated exceptional behavior, achieving complete MB removal within 30 min of reaction time and exhibiting outstanding stability and reusability after four reaction cycles. These findings highlight the potential of the synthesized compounds in the field of wastewater treatment for organic pollutants, offering a promising alternative to current environmental issues.

## Abstract 2

CdS-MIL-101 (Fe) composites exhibited remarkable stability and reusability. Even after four reaction cycles, they were capable of degrading 100% of methylene blue under UV and visible irradiation. The type I electronic band structure allowed the establishment of an efficient reaction mechanism that effectively inhibits the recombination of the photogenerated electron−hole pairs. It was found that ^•^O_2_^−^ radicals and holes are the main species involved in the photocatalytic degradation of methylene blue.

## 1 Introduction

Metal–organic frameworks (MOFs) are porous crystalline materials with large specific surface area, abundant active sites, and good thermal stability.^1^ MOFs can be described as organic–inorganic hybrid materials with intramolecular pores formed by the self-assembly of organic linkers and inorganic metal ions or groups through coordination bonds.^2^ MOFs have attracted great research interest in photocatalysis.^3,4^ Their high surface area, large porosity, and high adsorptive potential allow better energy absorption by the optically active sites.^5^ In particular, Fe-based or Fe-core MOFs can be considered viable photocatalysts because of their photosensitive and magnetic properties, low synthesis cost, and ease of reuse.^6^ An intense research study has been carried out regarding the Fe-based MIL series, for photocatalytic processes particularly addressed for the removal of water pollutants, which exhibited featured photocatalytic efficiency.^7,8^

The integration of MOFs with semiconductor photocatalysts can promote a positive effect by creating an advanced structure pursuing the enhancement of photocatalytic activity.^9^ This improvement is attributed to the unique heterojunction formed by a MOF-semiconductor structure that could promote the production of electron–hole (e^–^–h^+^) pairs, increasing the carrier migration, while reducing the recombination of the photogenerated pairs.^10,11^ In this regard, Wu et al.^12^ reported the synthesis of MIL-101(Fe)/Bi_2_WO_6_, where rhodamine B (RhB) and tetracycline hydrochloride (TC) were used as probe molecules to evaluate its photocatalytic capabilities. The experimental results showed that the photocatalytic degradation efficiency of the MIL-101 (Fe)/Bi_2_WO_6_ heterojunction under visible energy irradiation was 10 times higher than Bi_2_WO_6_. Hajiali et al.^10^ reported the integration of Bi_2_MoO_6_ with MIL-101 (Fe). This structure improved the photocatalytic activity to degrade RhB, reaching up to 85% removal under visible energy. The improvement in the photocatalytic response was attributed to the reduction of the band gap (*E*_g_) while decreasing the (e^–^–h^+^) recombination rate. In another study, Shi et al.^13^ reported the dispersion of CoFe_2_O_4_ nanoparticles on the MIL-101 (Fe) surface to degrade the TC compound under visible light irradiation. The compound was evaluated for 120 min, reaching 80% TC removal, demonstrating an excellent photocatalytic performance. The corresponding reaction rate constants were 3.3 and 14.9 times higher in comparison to MIL-101 (Fe) or CoFe_2_O_4_, respectively. The outstanding photocatalytic behavior was attributed to the synergistic effect of the CoFe_2_O_4_/MIL-101 (Fe) heterojunction.

Cadmium sulfide (CdS) exhibits outstanding optical and electrical properties^14^ being also an attractive option for photocatalytic applications.^14^ CdS is a type II–VI semiconductor with relatively narrow *E*_g_ (2.4 eV), which can be excited at a wavelength below 516 nm.^15^ Potential applications include photodetection,^16,17^ optoelectronic applications,^18,19^ energy storage,^20^ solar cells,^21^ and photocatalysis,^22,23^ among many others. However, CdS is susceptible to photocorrosion and rapid recombination of photoinduced (e^–^–h^+^) pairs under high-energy irradiation. To avoid those problems while improving transference of charge carriers,^24^ CdS is combined with other materials, including Cu-doped CdS nanoparticles,^25^ Au/CdS,^26^ CdS/ZnO nanorods,^27^ and CdS/ZnS.^28^ Considering these results, and the aforementioned properties of MOFs, and thus the integration of the CdS compound with a MOF structure, it is expected to exhibit a synergistic behavior enhancing its photocatalytic performance.^29^

For instance, Chen et al.^30^ reported the synthesis of CdS nanoparticles with graphitic carbon nitride (g-C_3_N_4_) sheets and a titanium metal–organic framework (MOF) using a facile solvothermal method, testing the catalytic photodegradation of rhodamine B (RhB) under visible light irradiation. The degradation rate reached 90% for RhB after 90 min of reaction, widely surpassing those of the CdS, MOF, and g-C_3_N_4_ photocatalysts under the same conditions. Jing et al.^31^ prepared a Cd-MOF, using it as a precursor to produce Cd-MOF/CdS nanocomposites through in situ sulfurization of Cd in the Cd-MOF. The degradation rate of MB under simulated sunlight irradiation was 91% in 100 min, which was higher than that of Cd-MOF (62%) or CdS (67%). Luo et al.^32^ prepared CdS@MOF-808 QD composites by a solvothermal method with the objective of suppressing the production rate of photogenerated carriers, testing it in the degradation of ciprofloxacin (CIP) under visible light irradiation. The composites exhibited a photocatalytic improved removal of about 82%, using CIP, compared to CdS and MOF-808. Jin et al.^33^ developed a hierarchical dual Z-scheme heterostructured photocatalyst prepared *via* an *in situ* hydrothermal synthesis method, where CdS nanoparticles were anchored onto the (UiO-66-NH_2_)-(MIL-101 (Fe)) (UM) dual metal–organic frameworks. Authors claim to have found a synergistic effect of CdS, UiO-66-NH_2_, and MIL-101(Fe), while the (UiO-66-NH_2_)-(MIL-101)(Fe)-CdS (UM-CdS) exhibits outstanding degradation activities toward TC. UM-CdS achieved a degradation value of 87% of TC in 140 min, which is 8.7, 2.4, and 1.4 times higher than those reached by UiO-66-NH_2_, MIL-101(Fe), and CdS, respectively. Zhang et al.^34^ developed a one-step solvothermal-precipitation strategy to prepare a novel Fe/Mn-MOF and CdS heterojunction bimetallic system. The bimetallic Fe/Mn-MOF@CdS catalyst exhibited enhanced activity for tetracycline removal (90%) compared to Fe/Mn-MOF (7%) and CdS (78%) after 160 min of reaction.

The performance of a material is not determined merely by its chemical composition; instead, it is also influenced by its structural, optical, and electrical characteristics. Among the methodologies for the production of MOF compounds, solvothermal^35,36^ and hydrothermal^37,38^ synthesis methods have proven to be effective. Recently, microwave-assisted hydrothermal methodology has shown technological advantages that greatly exploit the conventional hydrothermal synthesis with electromagnetic fields to directly convert microwaves into thermal energy. This method has become important because of its advantages over other synthesis methods. For instance, it allows for the rapid and uniform heating of the reactants. The heat is generated within the material instead of being absorbed from an external heat source. As a result, it creates a minimal overall temperature gradient in the material, which is influenced by factors such as dielectric properties, permittivity, and permeability that affect the distribution of the microwave electromagnetic field within the heating cavity and the depth of penetration.^39−41^ Additionally, it also promotes rapid nucleation, excellent particle control, high yields, reproducibility, short reaction times, and energy-saving.^42−45^

Based on the analysis described above, our work focuses on the synthesis and characterization of CdS, MOF MIL-101 (Fe), and CdS-MOF MIL-101 (Fe) compounds. CdS particles were integrated with different concentrations of MIL-101 (Fe) to achieve maximum efficiency in the photodegradation of MB. The synthesis was carried out by using a microwave-assisted method. Subsequently, the structural, morphological, textural, compositional, and optical properties of the materials were investigated through various characterization techniques. The synthesized compounds were subjected to a photocatalytic process to study the degradation of MB. The microwave-assisted synthesis provided an efficient photocatalytic system that produced a material suitable for the intended purpose. It was demonstrated through the proposed methodology that the outstanding performance of our catalysts shows superior degradation capability, achieving 100% degradation of MB while exhibiting high stability and reusability. The CdS-MOF heterojunction played a crucial role in the effective generation and transfer of electrons, which can be attributed to the synergistic effect between the MOF and CdS particles, which is reflected in its exceptional photocatalytic performance.

## 2 Experimental Section

### 2.1 Materials

Thiourea (CH_4_N_2_S, 99%) and cadmium acetate dihydrate (Cd(C_2_H_3_O_2_)_2_2H_2_O, 98%) from Sigma-Aldrich were used as sources of sulfur and cadmium, respectively. For the MOF MIL-101 (Fe), *N*,*N*-dimethylformamide (DMF, C_3_H_7_NO), terephthalic acid ((H_2_BDC, C_8_H_6_O_4_), 98%), iron(III) chloride hexahydrate (FeCl_3_·6H_2_O, 98%), and isopropyl alcohol (C_3_H_8_O) from Sigma-Aldrich were used. Deionized water was obtained by a LABCONCO WaterPro PS Polishing System. No further purification was required for all reagents.

### 2.2 CdS Synthesis

In a typical procedure, 20 mL of a thiourea solution (2 M) and 20 mL of a cadmium acetate solution (2 M) were mixed in a beaker and kept under stirring for 10 min. Then, the solution was transferred to a Teflon vessel, sealed, and placed in a microwave reactor. A heating ramp of 16 °C/min was employed until the temperature reached 160 °C, and it was maintained for 30 min under constant stirring. The power of the microwave reactor was 600 W. At the end of the reaction, the solution was cooled under room temperature conditions. The resulting orange precipitate was collected by centrifugation, washed with water and isopropyl alcohol, and dried at 120 °C for 12 h at 84,660 Pa.

### 2.3 MOF-MIL-101 (Fe) Synthesis

To synthesize the MOF MIL-101 (Fe), 0.7460 g of FeCl_3_·6H_2_O and 0.4584 g of H_2_BDC were mixed in 35 mL of DMF. The solution was stirred continuously for 10 min before being transferred to a Teflon vessel and then placed in the microwave reactor. The reactor was programmed with a 5 °C/min heating ramp until reaching 200 °C, and the temperature was maintained at 200 °C for 50 min under constant stirring. The resulting solution was centrifuged, and the precipitate was collected and poured into 30 mL of DMF. The solution was then stirred for 12 h, after which the product was collected. This procedure was repeated 3 times. Afterward, the product was washed with isopropyl alcohol and then with 30 mL of DMC. Finally, the resulting powder was dried at 120 °C for 12 h at 84,660 Pa.

### 2.4 CdS-MIL-101 (Fe) Synthesis

**Figure 1:**
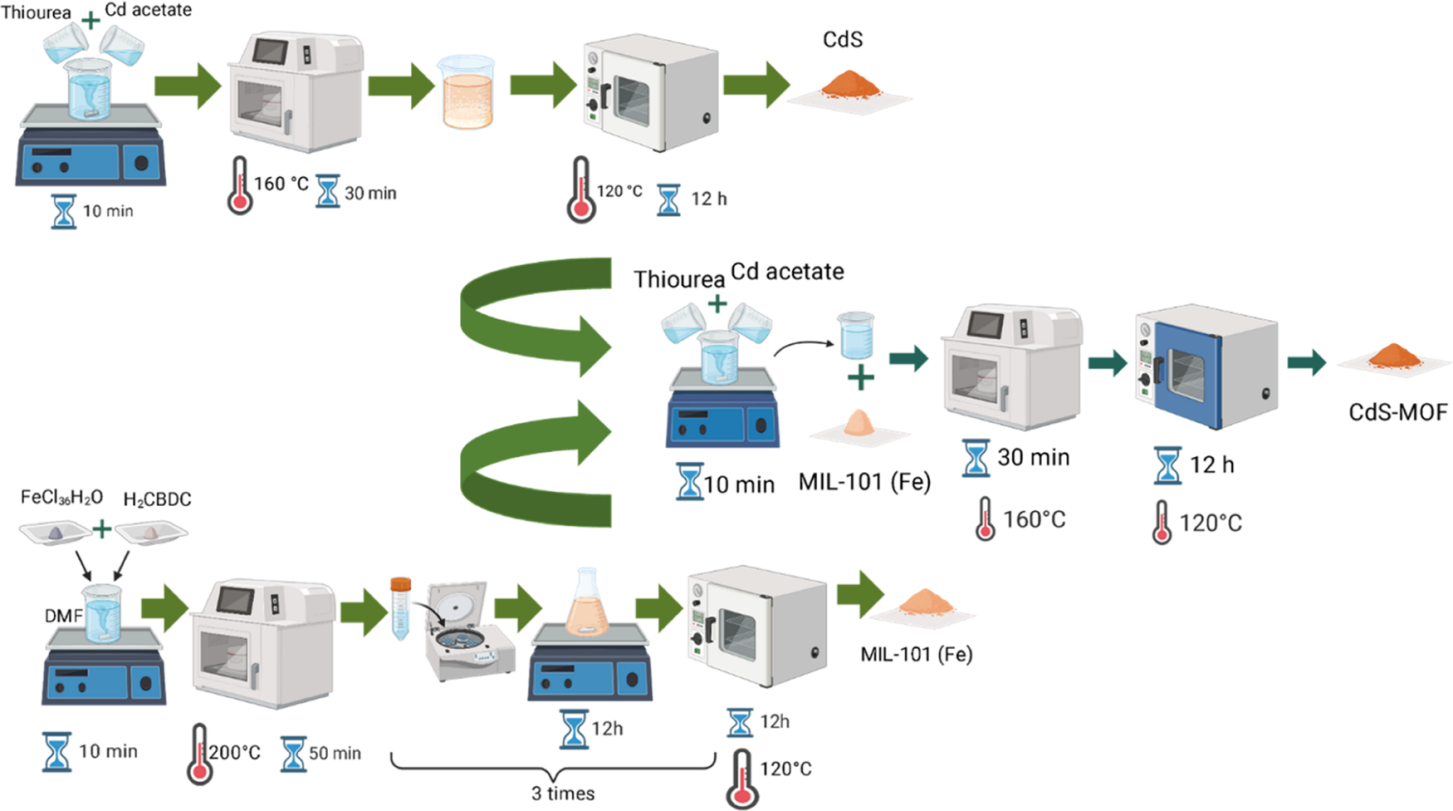
Representation of CdS, MIL-101 (Fe), and CdS-MOF synthesis. Created using BioRender software (http://biorender.com).

For the synthesis of the CdS-MOF MIL-101 (Fe) systems, 10 mL of a 2 M solution of thiourea and 10 mL of a 2 M solution of cadmium acetate were mixed in a beaker. The solution was stirred for 10 min and transferred to a Teflon vessel with 40 mg of MOF MIL-101 (Fe). A heating ramp of 16 °C/min was applied until the temperature reached 160 °C, and it was maintained for 30 min under constant stirring. The resulting precipitate was collected by centrifugation and subsequently washed with isopropyl alcohol and water and then dried in two stages. It was first dried at 100 °C for 24 h and, in a second stage, at 120 °C for 12 h at 84,660 Pa. Three additional syntheses were achieved with the purpose of changing the CdS-MIL-101 (Fe) ratio, adding 80 mg, 120 mg, and 160 mg of MOF MIL-101 (Fe) to the thiourea and cadmium acetate solution. The resulting compounds were labeled according to the amount of MOF 101 (Fe) added to the CdS: CdS-MOF-1, CdS-MOF-2, CdS-MOF-3, and CdS-MOF-4 for 40, 80, 120, and 160 mg, respectively. The synthesis was performed using a CEM MARS 6 Synthesis microwave reactor, and the synthesis process is summarized in Figure 1.

### 2.5 Characterization Techniques

X-ray diffraction patterns were obtained with a Bruker D-8 Advance diffractometer with monochromatic Cu–Kα radiation (λ = 1.5418 Å). The measurements were taken with a step time of 0.5 s, a step size of 0.02° (2θ), an excitation current of 30 mA, and a voltage of 40 keV. Morphological studies were conducted using a field emission scanning electron microscope (FE-SEM, JEOL JSM 7601F). Surface chemical composition studies were performed by using X-ray photoelectron spectroscopy (XPS). The XPS survey and XPS scans were obtained with Thermo Scientific K-α equipment equipped with an Al Kα monochromator. To ensure sample purity, erosion with argon was performed for 15 s. Raman spectra were obtained using a Witec 300 α confocal Raman spectrometer, with an excitation wavelength of 633 nm, 20 accumulations, and 2 s of integration time at a laser power of 10 mW. Infrared spectra were obtained using the Attenuated Total Reflection technique on Fourier transform infrared (FTIR) spectrometer (Thermo Scientific Nicolet iS5)., with a range of 4,000 to 500 cm^–1^, with 32 background scans and 8 resolutions as measurement conditions. The optical properties and degradation reactions were carried out with an AvaSpec-2048 spectrophotometer, which was equipped with a Mod Avalight-DH-S-BAL halogen-deuterium lamp and an Ocean Optics integrating sphere, Mod ISP-50–8-R-GT. Photoelectrochemical characterization was performed by using a three-electrode cell with a quartz window to allow sample illumination. MOF MIL-101 (Fe), CdS, and CdS-MOF samples were used as the working electrode (WE), a platinum wire was used as the counter electrode (CE), and a Ag/AgCl electrode was used as the reference electrode. The aqueous solution of KOH (1 M) was used as an electrolyte. The photovoltages and photocurrent densities were obtained under blue LED (455 nm) illumination. The measured light power density reaching the sample surface was 70 ± 5 mW/cm^2^. Electrochemical impedance spectroscopy (EIS) measurements were performed by using an Autolab PGSTAT302N/FRA II potentiostat (Metrohm) to obtain the flat band potentials (*V*_fb_) from the Mott–Schottky plot in the frequency range of 100–1000 Hz.

### 2.6 Degradation Studies

The synthesized photocatalysts were tested under both UV and visible irradiation. Each experiment required 20 mg of photocatalyst and 30 mL of MB at a concentration of 20 ppm. The catalyst concentration was 0.67 g/L. The solution containing the photocatalyst was stirred in the absence of light for 20 min to promote the adsorption–desorption equilibria between the solution and the photocatalyst surface. Then, the photocatalytic degradation process began. The samples were collected at specified intervals (0, 5, 15, 30, 60, 90, and 120 min) while the absorbance was measured at 664 nm for MB. The degradation percentage was calculated using the following equation:
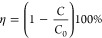
1where η is the percentage of degradation, *C*_0_ is the initial concentration, and *C* is the concentration after irradiation time. The reaction rate constants (κ) were calculated by using the first-order Langmuir–Hinshelwood kinetic model:
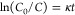
2For the UV degradation experiments, an Hg (Ar) lamp (Oriel 6035 model) was used, while a 50 W, 12 V halogen lamp was used for the visible degradation experiments.

## 3 Results

### 3.1 X-ray Diffraction

**Figure 2:**
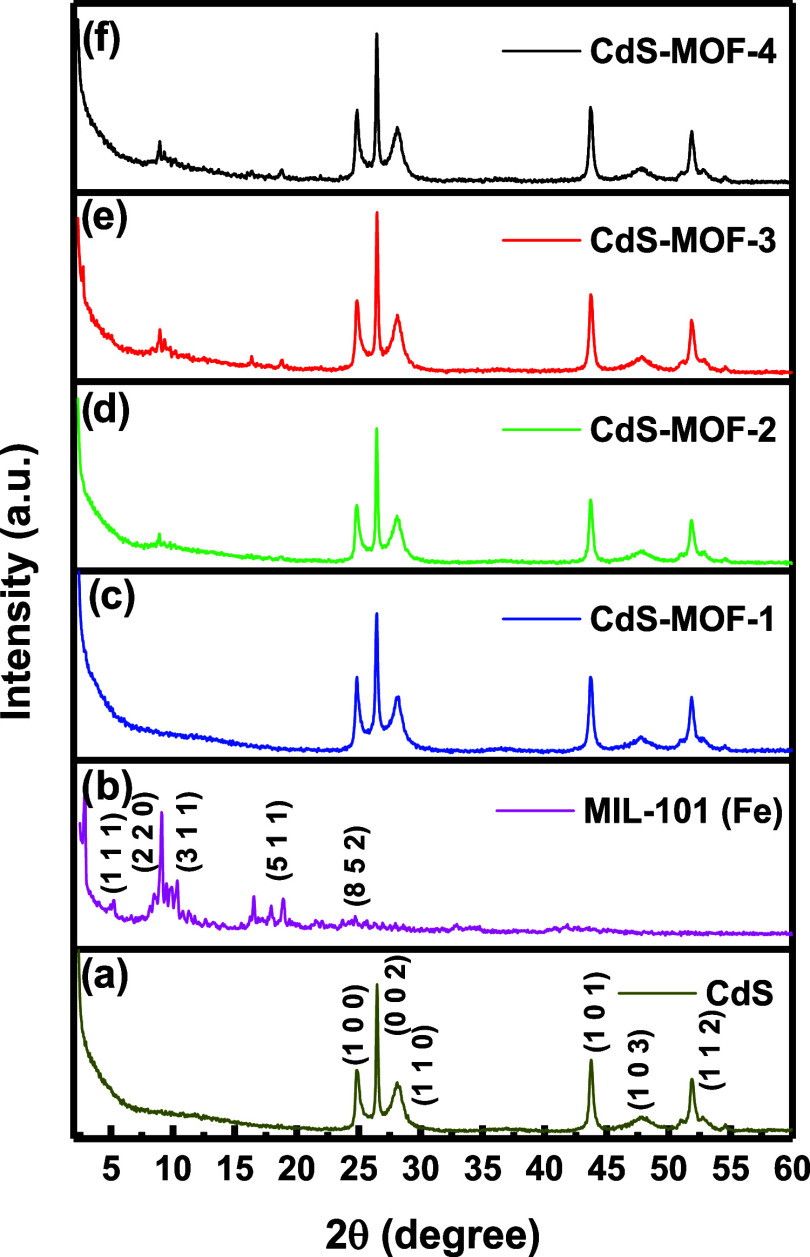
XRD patterns of (a) CdS, (b) MIL-101 (Fe), (c) CdS-MOF-1, (d) CdS-MOF-2, (e) CdS-MOF-3, and (f) CdS-MOF-4 compounds.

The X-ray diffraction patterns for the CdS, MIL-101 (Fe), and CdS-MOF systems are presented in detail in Figure 2. The diffraction measurements were performed over an angular range of 2θ = 3–60° (deg), providing comprehensive coverage for the identification of the characteristic peaks of each material. Figure 2a shows the diffraction pattern of CdS, where well-defined peaks can be observed at 2θ = 24.89, 26.47, and 28.10°. These peaks correspond to the crystal planes (100), (002), and (101), respectively. The presence of these peaks confirms the hexagonal crystal structure of CdS, as indicated by the diffraction data card JCPDS 77–2306, which is consistent with the data reported in the literature.^46,47^ On the other hand, Figure 2b illustrates the diffractogram of MIL-101 (Fe), which shows prominent peaks at 2θ = 5.18, 8.78, 9.34, 18.84, and 24.70°, corresponding to the crystal planes (111), (220), (311), (511), and (852), respectively. The obtained values are consistent with those reported in the literature,^35−37,48^ confirming the correspondence with the expected crystal structures. Furthermore, the analysis of the diffractograms for the CdS-MOF composites, shown in Figure 2c–f, reveals that the characteristic peaks of both MIL-101 (Fe) and CdS are present, demonstrating the success of the synthesis of the composite materials. Notably, as the concentration of the MOF in the compounds increased, the peaks in the diffractograms became more defined. This phenomenon indicates an improvement in the crystallinity of the CdS-MIL-101 (Fe) composites, proving the successful incorporation of CdS into the MOF.

### 3.2 Field Emission Scanning Electron Microscope and Energy-Dispersive Analyses

**Figure 3:**
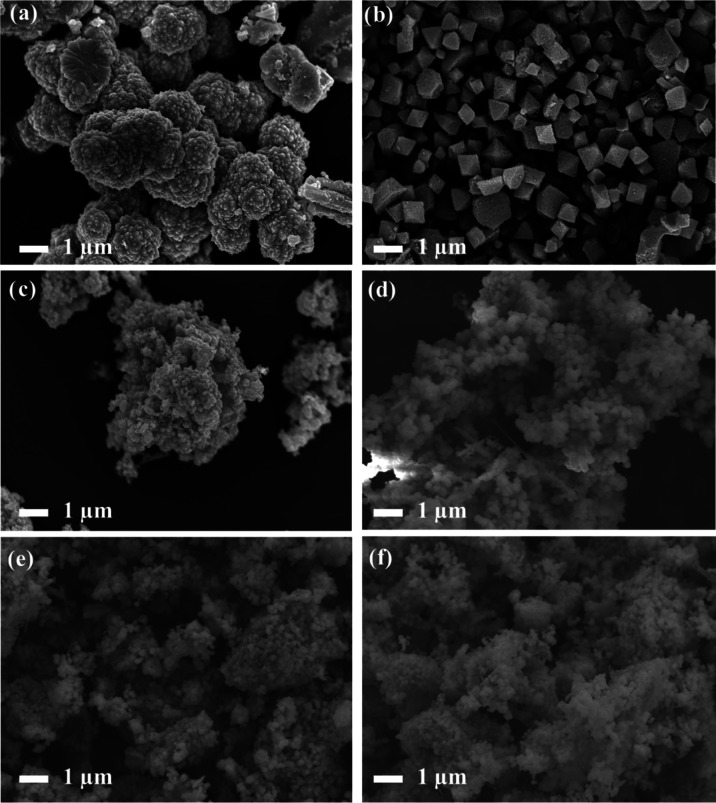
FE-SEM images of (a) CdS, (b) MIL-101 (Fe), (c) CdS-MOF-1, (d) CdS-MOF-2, (e) CdS-MOF-3, and (f) CdS-MOF-4 compounds.

Figure 3 shows the SEM micrographs for the different compounds. Figure 3a presents the SEM image of the CdS compound, revealing a tendency for particle aggregation during synthesis, forming spheres with a rough surface and sizes ranging from 1 to 2 μm. Figure 3b shows the SEM image of the MOF-MIL-101 (Fe) sample, which exhibits a uniform octahedral morphology with sizes of close to 1 μm. This morphology is consistent with previous reports,^46,47^ which describe MOF-MIL-101 (Fe) as a well-defined octahedral structure, indicating controlled and homogeneous growth of the material. Figure 3c–f shows the SEM images of the CdS-MOF compounds. The images reveal consistent morphologies, displaying aggregated particles of various sizes with rough surfaces. While these micrographs reveal the presence of CdS particles on the MOF, the underlying MOF structure cannot be clearly distinguished. This observation suggests that during the synthesis process, the octahedral MOF-MIL-101 (Fe) surface was enveloped by CdS particles. This behavior is consistent with the previously observed X-ray diffraction (XRD) patterns, which confirmed the incorporation of CdS onto the MOF surface without significantly altering its original octahedral structure.^49^

### 3.3 X-ray Photoelectron Spectroscopy

**Figure 4:**
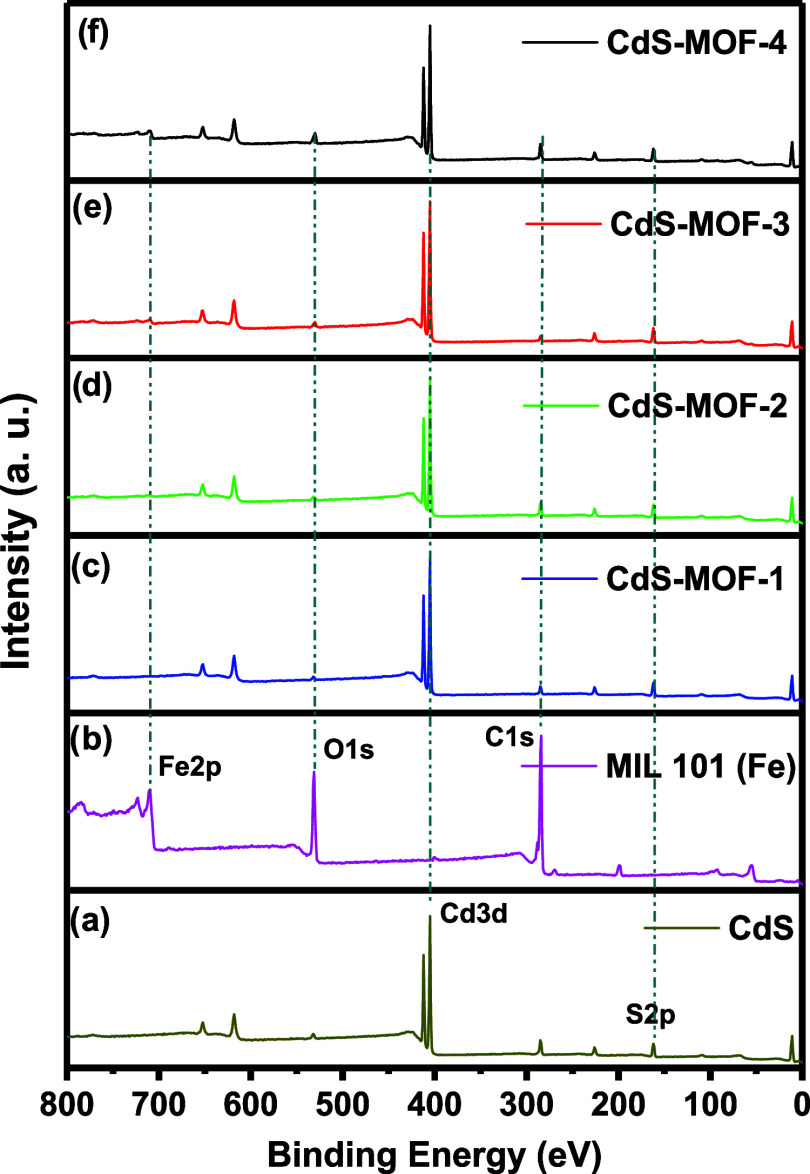
XPS survey for (a) CdS, (b) MIL-101 (Fe), (c) CdS-MOF-1, (d) CdS-MOF-2, (e) CdS-MOF-3, and (f) CdS-MOF-4 compounds.

XPS studies were conducted to confirm the presence of the principal elements in the samples. The XPS spectra for CdS, MIL-101 (Fe), and the CdS-MOF systems are presented in Figure 4, providing detailed insights into the elemental composition of each sample. For the CdS compound, the XPS spectra reveal the presence of the primary elements, Cd and S, confirming that these are the dominant components of the sample, with binding energies (BE) of 405.09 and 166.08 eV for the Cd 3d and S 2p peaks, respectively. In the case of the MIL-101 (Fe) sample, the XPS spectra displayed distinctive signals corresponding to carbon (C 1s), oxygen (O 1s), and iron (Fe 2p). The BE detected was 284.36 eV for C 1s, 531.19 eV for O 1s, and 739.08 eV for Fe 2p. These values are consistent with previous reports on the composition of MIL-101 (Fe).^4^ For the MOF-CdS systems, signals corresponding to Cd, S, C, O, and Fe were detected at BE of 406.06 eV for Cd, 161.98 eV for S, 284.85 eV for C, 530.4 eV for O, and 710.15 eV for Fe. The detection of these peaks at the expected energies confirms the effective integration and formation of each element into the CdS-MOF systems. These results provide additional evidence of the correct synthesis and structure of the analyzed materials.

**Figure 5:**
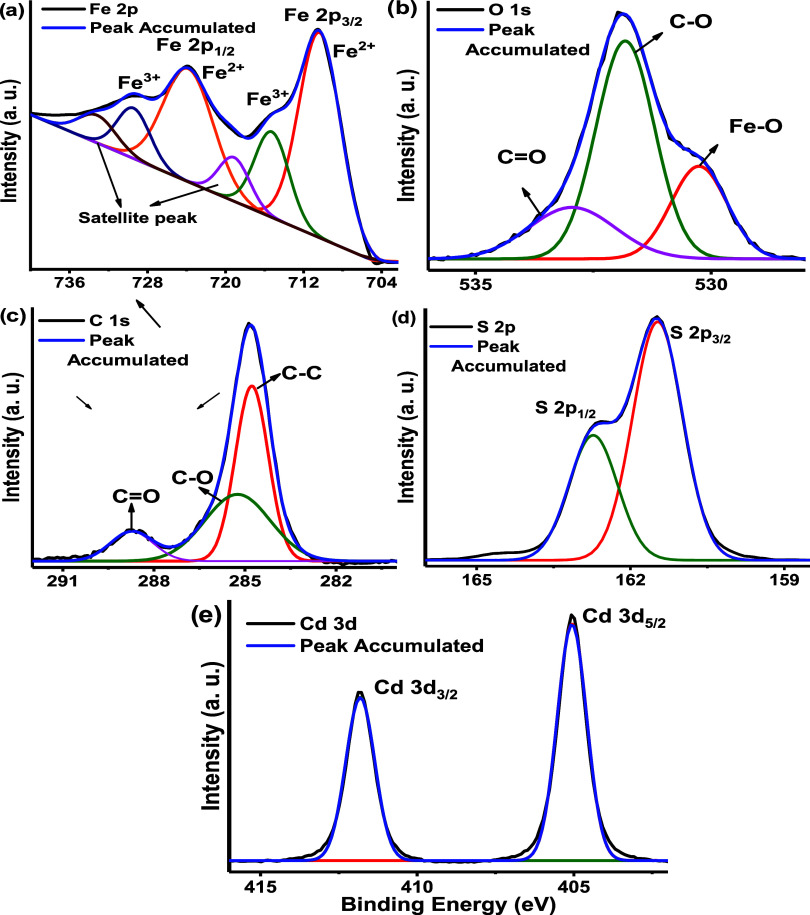
(a) HR-scan of the Fe 2p peak for MIL-101 (Fe), (b) HR-scan of the O 1s peak for MIL-101 (Fe), (c) HR-scan of the C 1s peak for MIL-101 (Fe), (d) HR-scan of the S 2p peak for CdS, and (e) HR-scan of the Cd 3d peak for CdS.

High-resolution (HR) XPS analysis was used to identify the different chemical species present in the compounds. Figure 5a−c shows the HR-scan peaks for MIL-101 (Fe). A deconvolution of the Fe 2p signal revealed the presence of six signals, including two strong peaks at 710.35 and 723.82 eV. These peaks can be attributed to the 2p_3/2_ and 2p_1/2_ orbits of Fe^2+^, respectively (Figure 5a). The peaks observed at 715.25 and 729.44 eV are attributed to the 2p_3/2_ and 2p_1/2_ orbits of Fe^3+^. Moreover, the peaks located at 719.12 and 733.15 eV are associated with satellite peaks of Fe^2+^ and Fe^3+^.^50^ The HR-scan of O 1s (Figure 5b) can be divided into three signals 530.25, 531.82, and 532.95 eV, attributed to oxygen atoms in the Fe–O, C–O, and C=O bonds, respectively.^12^ The HR-scan of C 1s is shown in Figure 5c. The successful synthesis of MIL-101 (Fe) is confirmed by the presence of three peaks, located at 284.77, 285.24, and 288.72 eV, which are related to (C–C) bonds of C atoms and organic carbon bonds (C–O and C=O).^51^Figure 5d depicts the HR-scan of S 2p corresponding to CdS. Two more peaks were observed at 161.42 and 162.72 eV, corresponding to S 2p_3/2_ and S 2p_1/2_, respectively, with an Δ*E* value of 1.23 eV, indicative of the formation of CdS.^47^ Finally, the HR-scan of Cd 3d (Figure 5e) revealed two peaks sited at 405.05 and 411.87 eV with Δ*E* = 6.72 eV, which were attributed to 3d_5/2_ and 3d_3/2_ of Cd^2+^, respectively.^52^

**Figure 6:**
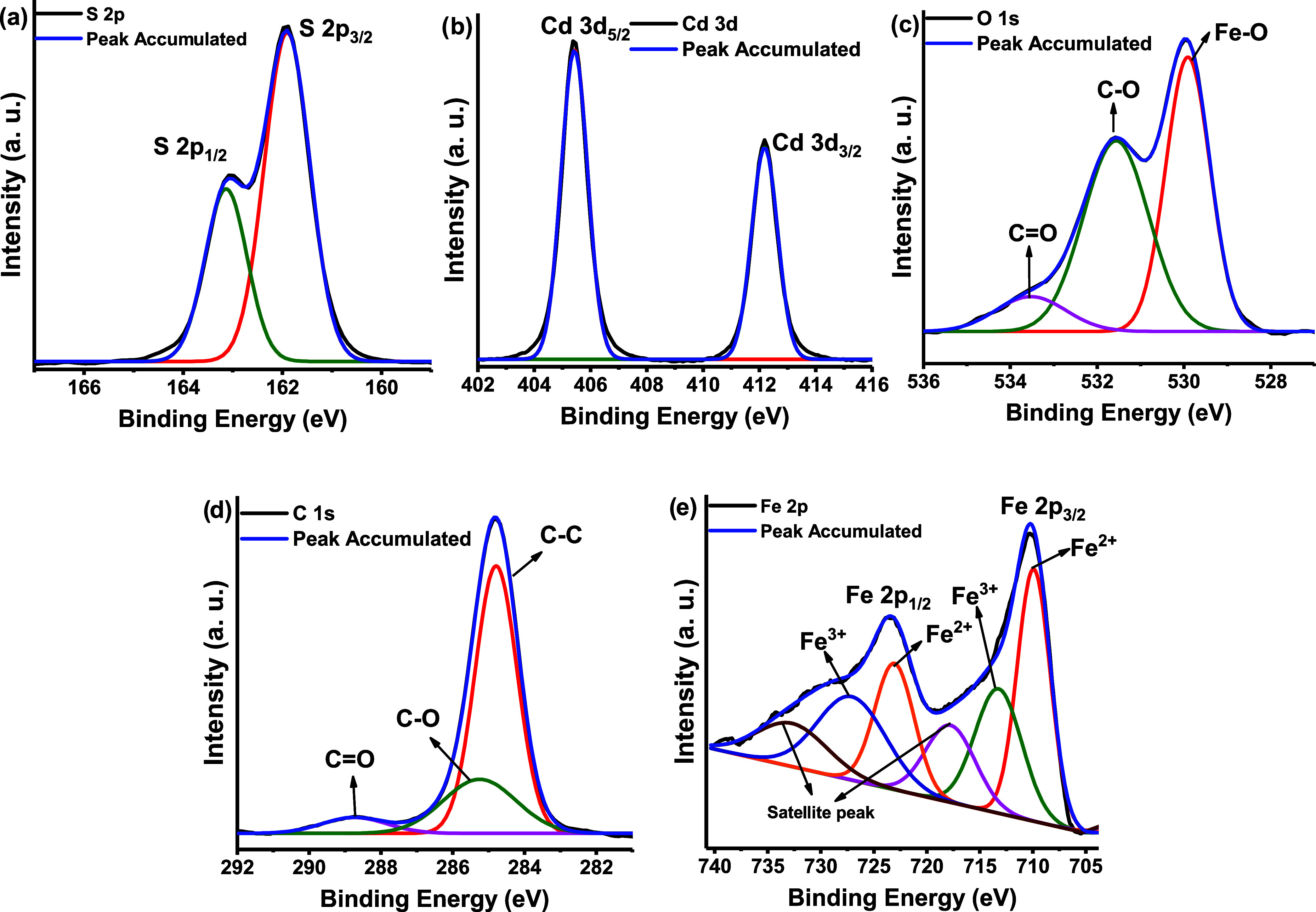
HR-scans for the CdS-MOF-3 compound. (a) HR-scan of the S 2p peak, (b) HR-scan of the Cd 3d peak, (c) HR-scan of the O 1s peak, (d) HR-scan of the C 1s peak, and (e) HR-scan of the Fe 2p peak.

Figure 6 presents the HR-scan for CdS-MOF-3. Figure 6a shows the HR-scan for S 2p. After performing the deconvolution process, two contributions were identified at 161.90 and 163.13 eV. These are related to the corresponding S 2p_3/2_ and S 2p_1/2_, respectively, with Δ*E* = 1.23 eV confirming the presence of S^2–^.^53^ Similarly, in Figure 6b, two signals were identified for Cd 3d located at 405.42 and 412.83 eV, corresponding to Cd 3d_5/2_ and Cd 3d_3/2_, respectively. These values are in close agreement with those reported in the literature for Cd^2+^ cations.^47,54^ The HR analysis of O 1s exposed in Figure 6c, revealed the presence of three distinct signals at 529.90, 531.56, and 533.52 eV, which correspond to Fe–O, C–O, and C=O bonds, respectively.^50^Figure 6d presents the HR-scan of C 1s, where three peaks were identified at 284.79, 288.73, and 285.25 eV, which are attributed to C–C, C–O, and C=O bonds, respectively.^12^Figure 6e presents an HR-scan of Fe 2p, which revealed six contributions, were detected. The 2p_3/2_ and 2p_1/2_ orbitals of Fe^2+^ were located at 709.90 and 723.04 eV, respectively. The values observed at 713.24 and 727.08 eV can be attributed to the 2p_3/2_ and 2p_1/2_ orbits of Fe^3+^, respectively. Finally, the peaks located at 717.76 and 732.72 eV are related to satellite peaks for Fe^2+^ and Fe^3+^, respectively.^35^ The formation of Fe^2+^ may be attributed to the departure of anionic ligands as a result of the synthesis conditions and the reduction of Fe^3+^ caused by DMF.^55,56^ Following this analysis, the successful synthesis of the CdS-MOF systems can be confirmed. Furthermore, it can be observed that the signals of the detected contributions exhibit a slight shift in their binding energies with respect to CdS and the MOF, which can be attributed to a chemically interactive interface among the compounds.^54^

### 3.4 UV–Vis Spectroscopy

**Figure 7:**
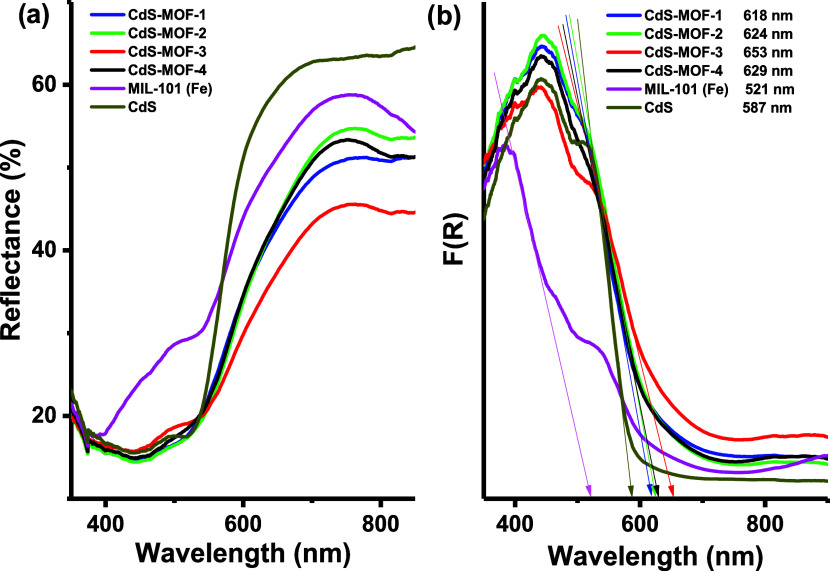
(a) UV–vis diffuse reflectance spectra and (b) absorption edge spectra using Kubelka–Munk approach.

The optical properties of the samples were analyzed by using UV–vis diffuse reflectance spectroscopy (DRS). Figure 7a shows the DRS spectra of CdS, MIL-101 (Fe), and the CdS-MOF composites. The spectra show that CdS and MIL-101 (Fe) exhibited a maximum value at 750 nm of about 65 and 60%, respectively. Similarly, the MOF exhibited a lower reflectance (30%) between 400 and 550 nm. Furthermore, it is noted that the incorporation of MIL-101 (Fe) into the CdS results in a decrease in the reflectance signal. These results suggest that the compounds can absorb and utilize irradiation within the visible region. The relationship between the absorption coefficient *K*, the scattering coefficient *S*, and the reflectance data *R* was calculated from the Kubelka–Mulk equation:^57^
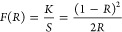
3The absorbance curves were plotted using eq 3 and are shown in Figure 7b. In MIL-101 (Fe), an intense absorption peak at 380 nm is observed, which is characteristic of the free organic linker, due to the π–π* and *n*–π* transitions.^58^ In contrast, from 450 nm and onward, MIL-101 (Fe) exhibits weak transitions, which are attributed to charge transfer in Fe–O groups of MOFs.^59^ In the synthesized CdS, the optical absorption edge was observed in the range of 440 and 570 nm, which corresponds to the shift from the valence band (BV) of S^2–^ ions to the conduction band (CB) of Cd^2+^ ions.^60^ The CdS and CdS-MOF composites showed very similar absorption in the range of 400–600 nm. However, the absorption edges of the CdS-MOF composites exhibited a slight red shift, indicating a reduction in the *E*_g_ compared to CdS and MOF. As illustrated in Figure 7b, the absorption band edge of CdS, MIL-101 (Fe), CdS-MOF-1, CdS-MOF-2, CdS-MOF-3, and CdS-MOF-4 is observed at 587.15, 521.22, 618.48, 624.82, 653.50, and 629.34 nm, respectively. To obtain *E*_g_ values, eq 4 was employed.
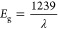
4The calculated *E*_g_ values are 2.11, 2.37, 2, 1.98, 1.89, and 1.96 eV for the CdS, MIL-101 (Fe), CdS-MOF-1, CdS-MOF −2, CdS-MOF-3, and CdS-MOF-4 compounds, respectively. The results demonstrate that the CdS-MOF compounds exhibited the lowest *E*_g_ value, which may contribute to the enhanced photocatalytic performance in the degradation of contaminants.

### 3.5 Raman Spectra

**Figure 8:**
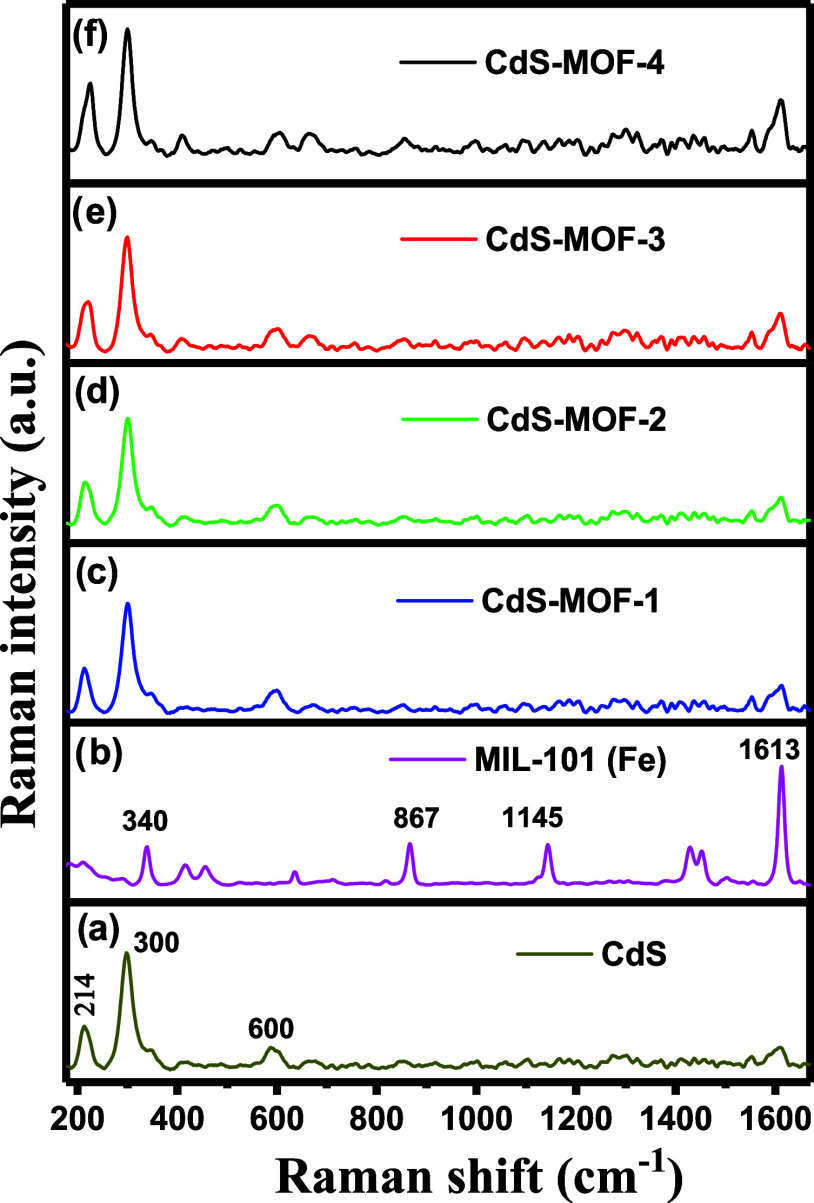
Raman spectra of (a) CdS, (b) MIL-101 (Fe), (c) CdS-MOF-1, (d) CdS-MOF-2, (e) CdS-MOF-3, and (f) CdS-MOF-4 compounds.

Figure 8a shows the Raman spectra obtained from the pristine CdS particles with peaks located at 214, 300, and 600 cm^–1^. The peaks located at 300 and 600 cm^–1^ are characteristic of the first (LO) and second (2LO) order longitudinal optical phonons, respectively.^61,62^ The peak observed at 214 cm^–1^, has been reported in works where the CdS nanoparticles depict sheet or wire-like morphologies.^63^ In our case, this can be attributed to the agglomeration of the rough CdS spheres. Figure 8b shows the Raman spectra for the MIL-101 (Fe) compound with characteristic peaks at 340 cm^–1^ attributed to the translational motions of the carboxyl ions. Additionally, bands at 867 and 1145 cm^–1^ can be related to the ring breathing of the MOF structure, while the peak at 1613 cm^–1^ is associated with the (COO^–^) antisymmetric stretching vibrations.^64−66^Figure 8c–f corresponds to the Raman spectra of the CdS-MOF systems, where the peaks corresponding to both materials can also be observed. However, in these cases, a blue shift occurs, probably due to the interaction caused by the impregnation of the CdS particles on the MOF surface, as demonstrated in the SEM images (see Figure 3).

### 3.6 FTIR Spectroscopy Studies

**Figure 9:**
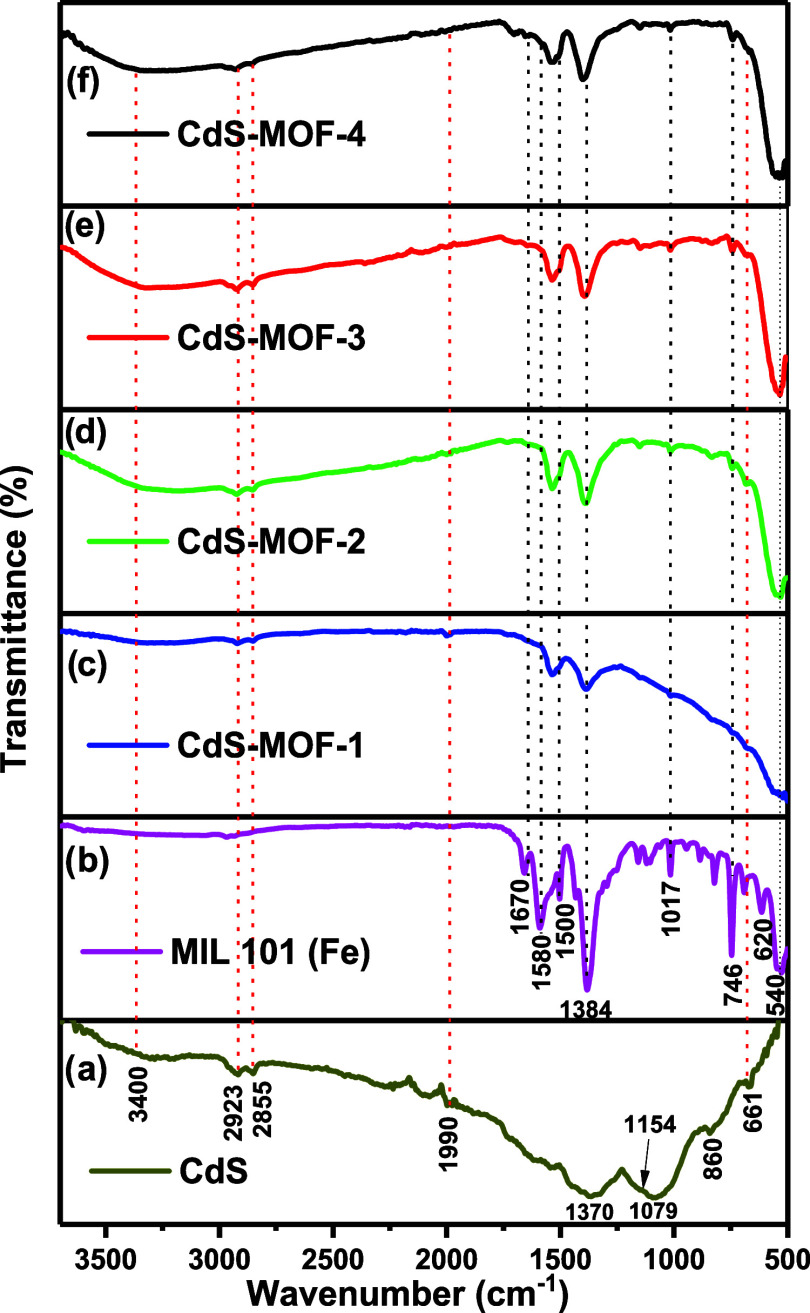
FTIR spectra of (a) CdS, (b) MIL-101 (Fe), (c) CdS-MOF-1, (d) CdS-MOF-2, (e) CdS-MOF-3, and (f) CdS-MOF-4 compounds.

The FTIR spectra in the range 500–3700 cm^–1^, of the synthesized compounds are presented in Figure 9. The spectrum of the CdS compound is displayed in Figure 9a. In this spectrum, the band appearing at 661 cm^–1^ is associated with the stretching of Cd–S; a frequency value that is usually detected below 700 cm^–1^. At higher wavenumbers, the bands appearing at 1079 and 1154 cm^–1^, correspond to the C–N/C=S stretch of thiourea. The band at 1370 cm^–1^ corresponds to the tris-amine C–N stretch shared with the C–O stretching. The band associated with N=C stretching, which is due to the hydrolysis of thiourea during the synthesis, appears at 1990 cm^–1^. The symmetrical and asymmetrical vibrations of the C–H group were also detected at 2855 and 2923 cm^–1^, respectively. Furthermore, the wavenumber bands at 860 and 3400 cm^–1^ were associated with the O–H stretching vibrations of H_2_O molecules.^67^ The FTIR spectrum of MIL-101 (Fe) is presented in Figure 9b. A broad band can be observed at 540 cm^–1^ that is related to Fe–O bonds.^10^ The band at 746 cm^–1^ is assigned to the C–H vibration of the benzene ring of H_2_BDC, while the bands at 1384 and 1580 cm^–1^ can be associated with the symmetric and asymmetric vibrations of O–C=C.^36^ The bands located at 1670 and 1500 cm^–1^ are assigned to the asymmetric stretching of C=O bonding carboxyl groups in H_2_BDC,^68^ and the band localized in 1017 cm^–1^ was assigned to C–O–C vibrations.^36^ Furthermore, the in-plane and out-of-plane bending modes of the COO groups could be detected for unreacted terephthalic acid inside the pores at 630 cm^–1^.^68^ Finally, the bending and stretching band between 3100 and 3400 cm^–1^ are assigned to the vibration of the water-absorbed O–H single bond on the sample surface.^3^ The FTIR spectra of the CdS-MOF composites are presented in Figure 9c–f. In the four spectra, the bands at 540 and 661 cm^–1^ associated with the Fe–O and Cd–S stretching can be observed. Likewise, the bands at 746, 1017, and 1348 cm^–1^ are related to the vibrations of C–H, C–O–C, and O–C=C, respectively. On the other hand, an overlap can be observed between the 1500 and 1580 cm^–1^ bands, from the C=O and O–C=C vibrations. These results indicate the proper formation of the synthesized compounds.

### 3.7 Photocatalytic Tests

**Figure 10:**
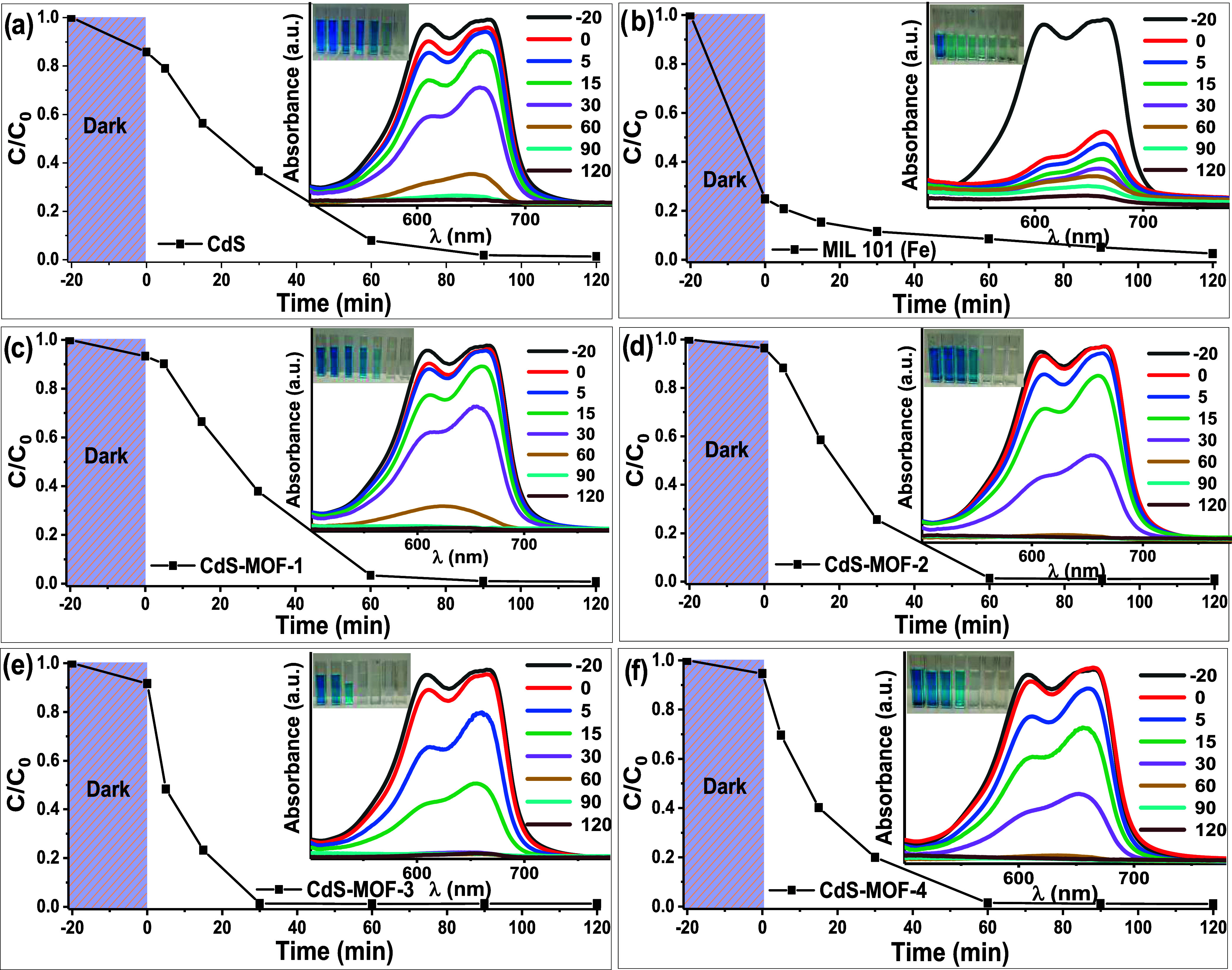
Photocatalytic degradation and MB absorbance profiles under UV irradiation using the (a) CdS, (b) MIL-101 (Fe), (c) CdS-MOF-1, (d) CdS-MOF-2, (e) CdS-MOF-3, and (f) CdS-MOF-4, compounds.

Figure 10 shows the degradation curves (*C*/*C*_0_) and absorbance profiles of MB using the different synthesized photocatalysts. The studies were carried out under UV energy (253 nm) irradiation for 120 min for every experiment. As described in Section 2.6, before each degradation test, the suspension was stirred homogeneously in the dark for 20 min to establish an adsorption–desorption equilibrium between the photocatalyst and the MB. After 20 min, the photocatalytic reaction began. Degradation percentages were calculated by using eq 1. The CdS compound showed an adsorption of 15% of MB in the first minutes to reach a degradation value of 100% in the subsequent 100 min. It was observed that MIL-101 (Fe) exhibited a strong adsorption of the dye, close to 76%, with a photocatalytic degradation value of 98% after 120 min, which was slower compared to the value for CdS. Subsequently, an improvement in the photocatalytic activity for the CdS-MOF systems was verified, in comparison to MIL-101 (Fe) and CdS. According to the results shown in Figure 10c,f, the CdS-MOF systems showed lower adsorption, close to 8%, indicating that the photocatalytic process was predominant. The results show that the CdS-MOF-3 compound exhibited the best photocatalytic response, reaching 100% of dye removal, after approximately 30 min of reaction, followed by the CdS-MOF-2, CdS-MOF-4, and CdS-MOF-1. All of them reach 100% of MB degradation at different time values of 60, 70, and 90 min, respectively. The results found, show that the combination of MIL-101 (Fe) and CdS achieves a synergistic effect that could be attributed to the dispersion of particles, the increase in h^+^ necessary for e^–^ transfer, and/or the inhibition of the recombination of the (e^–^–h^+^) pair, improving thus the photocatalytic efficiency.^12^

**Figure 11:**
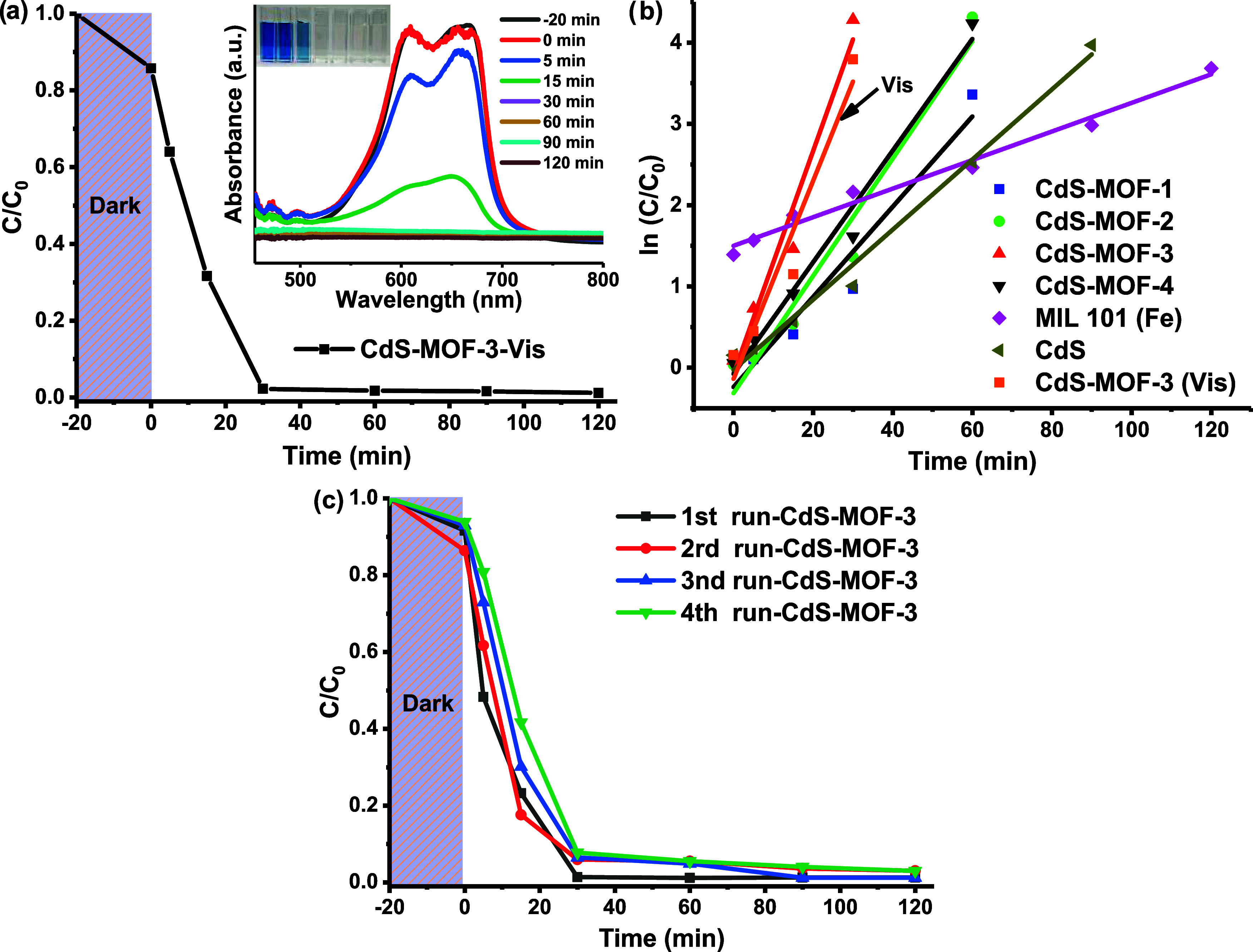
(a) Photocatalytic degradation and MB absorbance profiles under visible irradiation using the CdS-MOF-3. (b) First-order reaction kinetic curves for CdS, MIL-101 (Fe), CdS-MOF-1, CdS-MOF-2, CdS-MOF-3, CdS-MOF-4 compounds under UV irradiation, and CdS-MOF-3 under visible irradiation. (c) Stability tests for MB degradation using the CdS-MOF-3 compound under UV irradiation.

The CdS-MOF-3 compound exhibited the best photocatalytic performance for the MB molecule under UV irradiation. To test the photocatalyst against the same dye under visible energy irradiation, similar experimental conditions were maintained and the degradation profile is presented in Figure 11a. The results showed that the CdS-MOF-3 compound can also act as a photocatalyst after testing the MB molecule under visible energy irradiation. In fact, the compound achieved 100% degradation in about 30 min of reaction. The kinetic studies (Figure 11b) indicated that the photocatalytic reactions follow a first-order kinetics, where the *R*^2^ correlation coefficients range between 0.94 and 0.98. The *k* values for CdS and MIL-101 (Fe) were 0.0432 and 0.0176 min^–1^, respectively. The results demonstrated that by combining CdS and MIL-101 (Fe), the systems obtained present an increase in the value of *k*. The CdS-MOF-3 compound presented the highest *k* result, with a value of 0.1371 min^–1^, which is approximately 3 and 7 times higher than those of CdS and MIL-101 (Fe), respectively. Besides, for the test performed under visible energy, the CdS-MOF-3 compound presented a constant *k* = 0.1226 min^–1^, which is slightly lower than that obtained for the same compound under UV energy irradiation. The results are summarized in Table 1. In a subsequent stage, considering that the CdS-MOF-3 compound exhibited the best photocatalytic performance, it was chosen to achieve stability tests, which were carried out under UV energy irradiation. The degradation rate of MB was about 90% after four reaction cycles (Figure 11c), indicating that the material preserves its photocatalytic features. The marginal decrease in photocatalytic performance could be attributed to partial blocking of the active sites or loss of active functional groups on the photocatalyst surface.^12^

**Table 1:** Rate Constants from MB Degradation Studies

	adsorption		photodegradation			
compound	%	time (min)	%	time (min)	κ	*R*^2^
**CdS**	15	20	83	120	0.0432	0.9897
**MIL-101 (Fe)**	75	20	23	120	0.0176	0.9831
**CdS-MOF-1**	7	20	93	60	0.0555	0.9454
**CdS-MOF-2**	4	20	96	60	0.0722	0.9595
**CdS-MOF-3**	9	20	91	30	0.1371	0.9638
**CdS-MOF-4**	6	20	92	60	0.0687	0.9897
**CdS-MOF-3-Vis**	15	20	85	30	0.1226	0.9451

**Figure 12:**
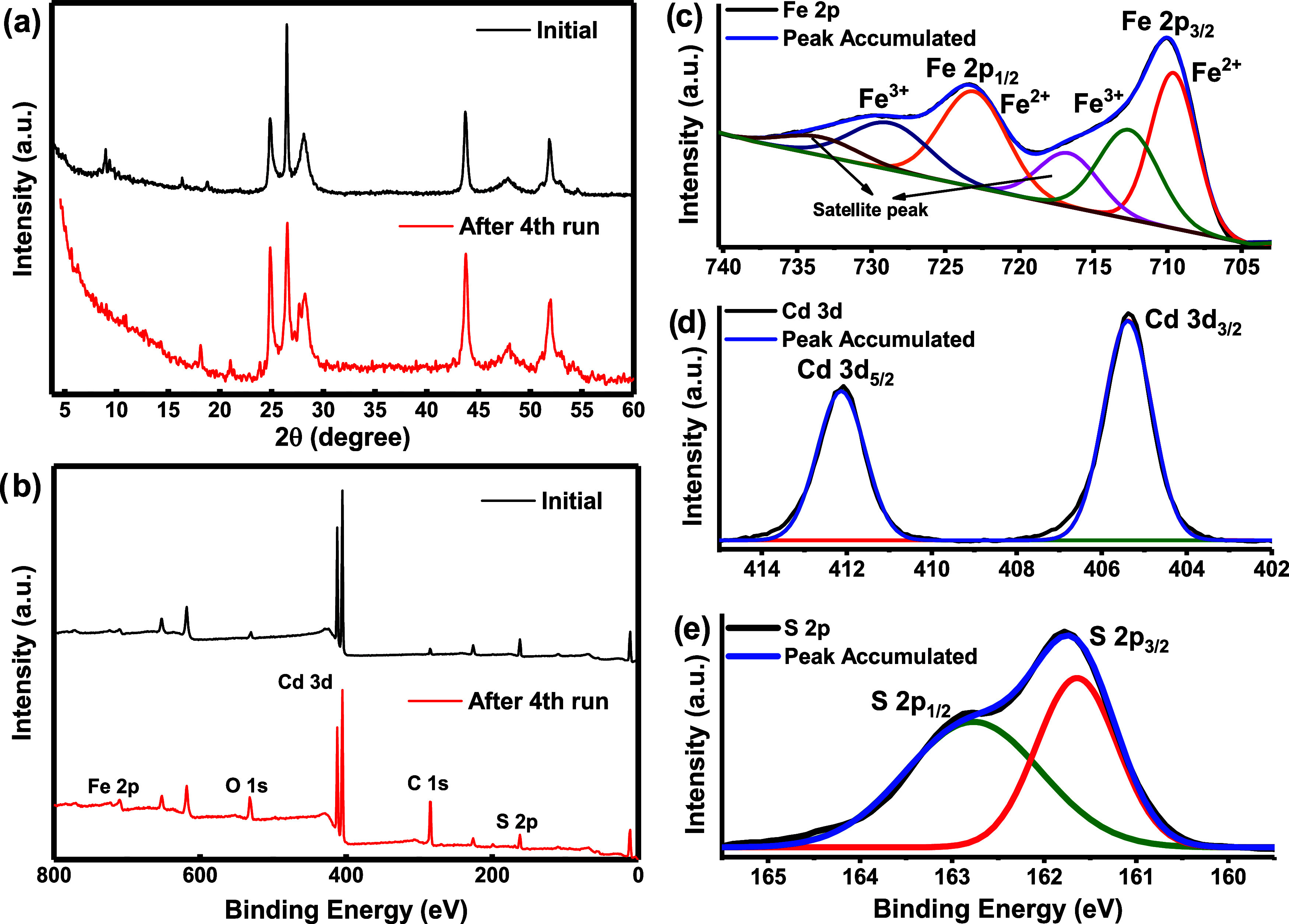
Stability CdS-MOF-3 after four cycles of photocatalytic activity in the elimination of MB. (a) X-ray diffraction patterns, (b) XPS survey, (c) HR-scan of the Fe 2p peak, (d) HR-scan of the Cd 3d peak, and (e) HR-scan of the S 2p peak.

The XRD patterns and the XPS survey of CdS-MOF-3 were compared before and after stability tests. XRD (Figure 12a) showed that the crystal structure of the composite does not change significantly and maintains its initial crystal structure. XPS survey spectra analyses of CdS-MOF-3 were carried out after achieving four degradation tests showing the presence of the Cd, S, Fe, O, and C elements (Figure 12b). The chemical composition (atom %) determined for the CdS-MOF-3 compound; before and after the reaction tests is shown in Table 2, where an increase in the C element content is observed, which goes from 40.7 to 56.7%, and a relative decrease in the Fe, O, Cd, and S elements, due to the increase in the percentage of C. This increase in the C content could be attributed to the fouling of the composite surface by reaction byproducts. This explains the marginal decrease in the photocatalytic performance after each reaction cycle. The HR-scans of Fe 2p, Cd 3d, and S 2p after four reaction cycles are presented in Figure 12c–e. In the HR-scans, the representative signal of each element can be observed. Therefore, the synthesized CdS-MIL-101 (Fe) composite demonstrates good stability, overcoming the observed limitations of pristine CdS, which is vulnerable to photocorrosion.

**Table 2:** Atomic Percentage Values from XPS Analysis for CdS-MOF-3 after Four Cycles of Photocatalytic Testing of MB

	element (atom %)				
	Cd	S	Fe	C	O
**CdS-MOF-3-Initial**	19.2	17.9	6.9	40.7	15.2
**CdS-MOF-3-After**	12.2	10.9	4.15	56.7	16

Table 3 presents the photocatalytic activity of some photocatalysts reported in the literature. Table 3 compares the concentration of MB (ppm), the dose of the photocatalyst (mg), the reaction time (min), the percentage of degradation, and the irradiation source. Although the initial conditions are different from those used in this work, it can be observed that in the present case, the percentage of MB removal reached 100% in 30 min when using 20 mg of the CdS-MOF-3 photocatalyst, which indicates the high competitiveness of our synthesized compounds.

**Table 3:** Comparative Analysis of the MB Photodegradation Performance under Several Catalysts and Conditions

compound	volume, MB concentration	catalyst dosage (mg)	time (min)	degradation (%)	light source	refs
**Ni-TiO**_**2**_	100 mL, 10 ppm	100	150	36	vis	(69)
**BiVO**_**4**_	20 mL, 20 ppm	10	80	86	sunlight	(70)
**Ce dopedZnO:TiO_2_**	50 mL, 10 ppm	30	90	98	vis	(71)
**MIL-101 (Fe)/Ce/g-C**_**3**_**N**_**4**_	50 mL, 15 ppm	15	75	88	vis	(72)
**S-doped-*g*- C**_**3**_**N**_**4**_**/MIL-101 (Fe)**	200 mL, 10 ppm	20	120	83	vis	(73)
**MIL-101 (Fe)/Bi**_**2**_**WO**_**6**_**/Fe (III)**	100 mL, 20 ppm	50	75	78	vis	(74)
**MoS**_**2**_**/MIL-101 (Fe)/carboxylated**	50 mL, 20 ppm	180	24	93	vis	(75)
**TiO**_**2**_**/g-C**_**3**_**N**_**4**_	50 mL, 10 ppm	10	40	100	vis	(76)
**CeO**_**2**_**/MnFe**_**2**_**O**_**4**_	25 mL, 25 ppm	3	180	86	vis	(77)
**NiFe**_**2**_**O**_**4**_	50 mL, 50 ppm	40	150	99	vis	(78)
**CdS-MOF-3**	30 mL, 20 ppm	20	30	100	UV	this work
**CdS-MOF-3**	30 mL, 20 ppm	20	30	100	vis	this work

**Figure 13:**
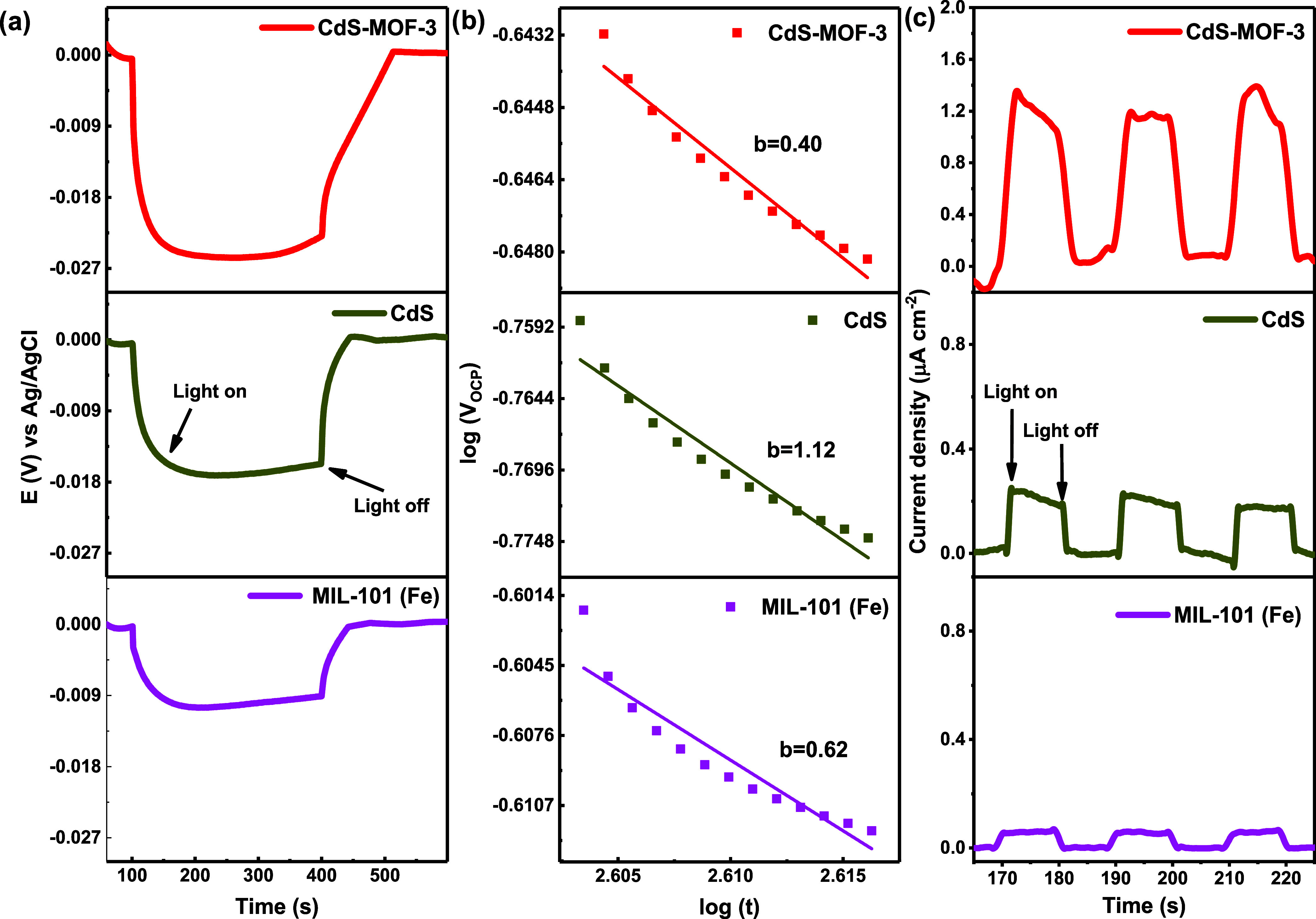
(a) Variation of *V*_OCP_ as a function of time, in the presence and absence of illumination, CdS, and CdS-MOF-3 compounds, and (b) plot of log(*V*_OCP_) vs log(*t*) for photovoltaic decay curve for CdS, and CdS-MOF-3 compounds. (c) Photocurrent response for MIL-101 (Fe), CdS, and CdS-MOF-3 compounds.

In order to provide a deeper understanding of the results obtained on the photocatalytic activity of the materials developed in the present work, a characterization procedure was carried out in PEC using a 455 nm LED (blue light) lamp in order to obtain the photo response of the materials (photovoltage and photocurrent density). The variation of *V*_OCP_ with respect to time for the MIL-101 (Fe), CdS, and CdS-MOF-3 compounds is presented in Figure 13a. When the light is turned off, e^–^ recombine causing the time variation of the voltage *V*_OCP_(*t*), which tends asymptotically toward less negative potential values until an equilibrium is established in darkness. This behavior can be represented by the equation:^79^
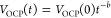
5where *V*_OCP_(0) is the open-circuit potential at equilibrium during illumination, *t* is the time elapsed since the light is turned off, *V*_OCP_(*t*) is the open-circuit potential at each time *t*, and *b* is a constant that can be associated with the recombination of electrons. The recombination constants *b*, calculated from the slopes of Figure 13b, were 0.62, 1.12, and 0.40 for MIL-101 (Fe), CdS, and CdS-MOF-3, respectively, which shows that the (e^–^–h^+^) rate recombination rate is lower for the CdS-MOF-3 compound.

An important test to contribute to the understanding of charge separation efficiency in photocatalysts and photocurrent density (transient and stationary) data is the chronoamperometry, which is performed through several cyclic on–off events. The results are presented in Figure 13c. As can be seen, the compounds exhibit an approximately stable photocurrent response during the 10 s on–off cycles. An important result is that the photocurrent density produced by the CdS-MOF-3 compound is 5.4 times higher than that of CdS, demonstrating a faster separation efficiency of the photoinduced (e^–^–h^+^) pairs in this compound; consequently, it is possible to expect an improved photocatalytic behavior.

**Figure 14:**
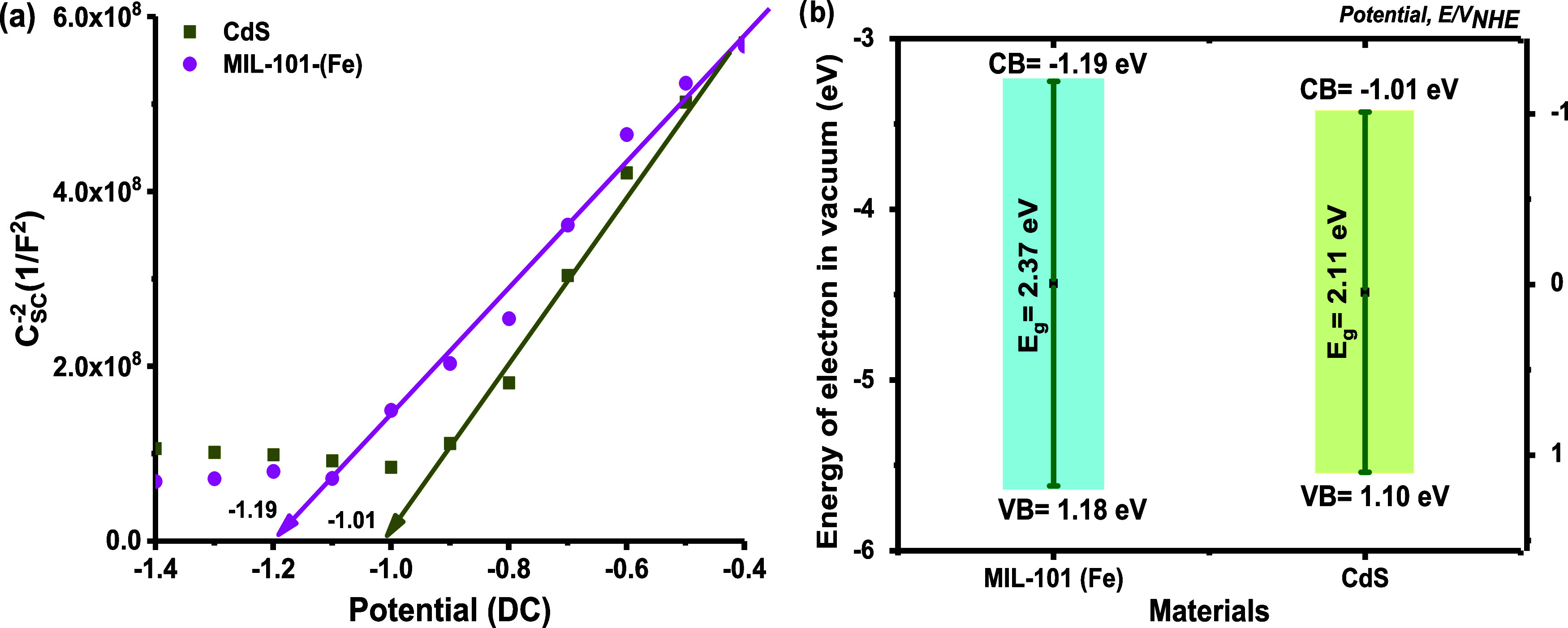
(a) Mott–Schottky electrochemical plots and (b) electronic band structure of MIL-101 (Fe) and CdS.

To explore the possible photocatalytic mechanism, Mott–Schottky diagrams were obtained to determine the flat band potential (*V*_fb_) and establish the electronic band structures. *V*_fb_ was obtained from the space charge layer capacitance, *C*_sc_, which was calculated from the imaginary part of the impedance (*Z*_im_), using eq 6:
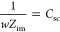
6where *w* is the frequency. To determine *C*_sc_, AC modulated cyclic voltage scans from −1.5 to 0.3 V were performed at frequencies ranging from 100 to 1000 Hz. The relationship between *C*_sc_ and the bias potential (*V*) is described by the Mott–Schottky equation:
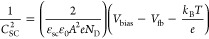
7where *A* is the WE area, *e* is the electronic charge, ε_sc_ is the dielectric constant of the material, ε_0_ is the permittivity of vacuum, *N*_D_ is the number of donors, *V*_bias_ is the applied bias potential in volts, *k*_B_ is the Boltzmann constant, and *T* is the temperature in absolute units (298 K). Accordingly, a plot of *C*_SC_^–2^ versus applied potential (*V*) should yield a straight line from which flat band potential can be determined from the intercept on the *V* axis.^80^ The calculated *V*_fb_ values for CdS and MIL-101 (Fe) were −1.01 and −1.19 V (vs Ag/AgCl), respectively. As shown in Figure 14a, the compounds showed a positive slope, indicating that they are n-type semiconductors; therefore, the conduction potential (*E*_CB_) is approximately 0.20 eV smaller than their *V*_fb_.^81,82^ Thus, the calculated *E*_CB_ values were −1.21 V (CdS) and −1.39 V (MIL-101 (Fe)). The *E*_CB_ vs normal hydrogen electrode (NHE) was calculated from the following equation:^83^
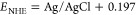
8The resulting valence band potentials (*E*_NHE_) were −1.01 and −1.19 eV for CdS and MIL-101 (Fe), respectively. From the results obtained for the *E*_g_ energy and using eq 9, the *E*_VB_ value was calculated. The *E*_VB_ values were 1.10 and 1.18 eV for CdS and MIL-101 (Fe) (vs NHE). The electronic band structure is presented in Figure 14b.
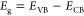
9

**Figure 15:**
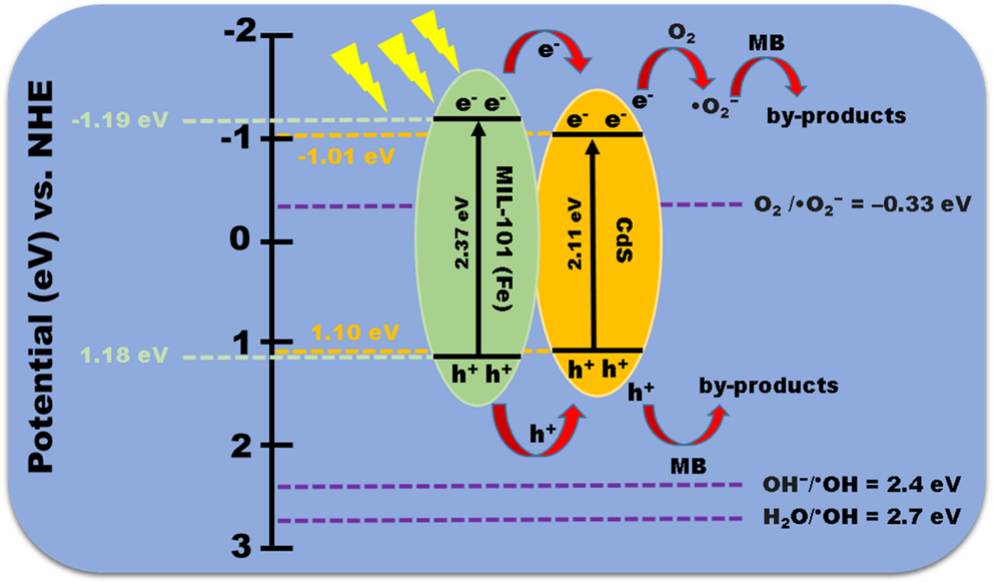
Mechanisms of photocatalytic activity of a type I heterojunction of CdS-MIL-101 (Fe).

According to the results, a charge transfer mechanism of electrons (e^–^) and holes (h^+^) is proposed for a better understanding of the MB degradation process in our experiments (Figure 15). The heterostructure composed of CdS and MIL-101 (Fe) corresponds to a type I heterojunction photocatalytic system, i.e., the CB and VB edges of CdS are overlapped with the *E*_g_ of MIL-101 (Fe).^84^ In this system, under energy irradiation, both MIL-101 (Fe) and CdS absorb energy to produce (e^–^–h^+^) pairs (eqs 10 and 11). Due to the VB potentials, the h^+^ accumulated in the VB of MIL-101 (Fe) and CdS does not have enough energy to oxidize H_2_O or OH^–^ to generate ^•^OH (OH^–^/^•^OH = 2.4 eV and H_2_O/^•^OH = 2.7 eV vs NHE);^85^ therefore, the ^•^OH radical is not the main reactive species specie in the degradation mechanism. On the other hand, the CB potential of MIL-101 (Fe) is more negative than the CB potential of CdS, and the VB potential of MIL-101 (Fe) is more positive than the VB potential of CdS; consequently, the e^–^ and the h^+^ in MIL-101 (Fe) can be transferred by the contact electric field to the CB and VB of the CdS, respectively, resulting in an efficient separation of the photoexcited (e^–^–h^+^) pairs (eq 12). Because the CB potential of CdS is more negative (−1.013 eV) than the potential of O_2_/^•^O_2_^–^ = −0.33 eV vs NHE,^86^ the process of O_2_ reduction to generate ^•^O_2_^–^ radicals can be achieved (eq 13). The ^•^O_2_^–^ radicals can degrade MB, while h^+^ can directly oxidize MB to produce byproducts (eqs 14 and 15).^84^
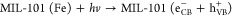
10
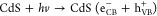
11
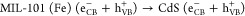
12
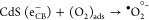
13
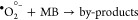
14
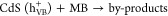
15

## 4 Conclusions

CdS-MIL-101 (Fe) compounds have been successfully synthesized by using a microwave-assisted heating method. CdS was integrated with varying concentrations of MIL-101 (Fe) to achieve maximum efficiency in MB photodegradation. The characterizations performed confirmed the formation and enhanced physicochemical properties of the heterojunction between CdS and the MOF MIL-101 (Fe). UV–vis analyses indicated a reduction in the *E*_g_. Transient photocurrent studies revealed a stronger photocurrent intensity, demonstrating improved separation efficiency of the photoinduced (e^–^–h^+^) pairs of CdS-MIL-101 (Fe) composites compared with CdS or MIL-101 (Fe) alone. Open-circuit potential (*V*_ocp_) studies showed that charge carrier recombination is lower in the CdS-MOF-3 compound. This novel photocatalytic system exhibited excellent stability and reusability, achieving 100% degradation of the MB after four reaction cycles. The type I electronic band structure allowed for the establishment of an efficient reaction mechanism that effectively inhibits the recombination of the photogenerated (e^–^–h^+^) pairs. Additionally, it was found that the ^•^O_2_^–^ radical and h^+^ are the primary species involved in the photocatalytic degradation of MB. The microwave-assisted synthesis produced an efficient photocatalytic system, resulting in a material well suited for its intended purpose. The results demonstrate that CdS-MOF compounds exhibit promising physicochemical characteristics, making them suitable for various technological applications such as the removal of organic contaminants from wastewater.
